# The global patent landscape of emerging infectious disease monkeypox

**DOI:** 10.1186/s12879-024-09252-w

**Published:** 2024-04-15

**Authors:** Yuanqi Cai, Xiaoming Zhang, Kuixing Zhang, Jingbo Liang, Pingping Wang, Jinyu Cong, Xin Xu, Mengyao li, Kunmeng Liu, Benzheng Wei

**Affiliations:** 1 Center for Medical Artificial Intelligence, Qingdao Academy of Chinese Medical Sciences, Shandong University of Traditional Chinese Medicine, 266112 Qingdao, China; 2 Department of Cardiovascular Surgery, The Affiliated Hospital of Qingdao University, 266000 Qingdao, China; 3 Department of Biomedical Sciences, Jockey Club College of Veterinary Medicine and Life Sciences, City University of Hong Kong, Kowloon Tong, Hong Kong SAR China; 4 State Key Laboratory of Quality Research in Chinese Medicine, Institute of Chinese Medical Sciences, University of Macau, 999078 Taipa, Macau China

**Keywords:** Monkeypox, Infectious disease, Patent landscape, Network analysis, Global trends

## Abstract

**Background:**

Monkeypox is an emerging infectious disease with confirmed cases and deaths in several parts of the world. In light of this crisis, this study aims to analyze the global knowledge pattern of monkeypox-related patents and explore current trends and future technical directions in the medical development of monkeypox to inform research and policy.

**Methods:**

A comprehensive study of 1,791 monkeypox-related patents worldwide was conducted using the Derwent patent database by descriptive statistics, social network method and linear regression analysis.

**Results:**

Since the 21st century, the number of monkeypox-related patents has increased rapidly, accompanied by increases in collaboration between commercial and academic patentees. Enterprises contributed the most in patent quantity, whereas the initial milestone patent was filed by academia. The core developments of technology related to the monkeypox include biological and chemical medicine. The innovations of vaccines and virus testing lack sufficient patent support in portfolios.

**Conclusions:**

Monkeypox-related therapeutic innovation is geographically limited with strong international intellectual property right barriers though it has increased rapidly in recent years. The transparent licensing of patent knowledge is driven by the merger and acquisition model, and the venture capital, intellectual property and contract research organization model. Currently, the patent thicket phenomenon in the monkeypox field may slow the progress of efforts to combat monkeypox. Enterprises should pay more attention to the sharing of technical knowledge, make full use of drug repurposing strategies, and promote innovation of monkeypox-related technology in hotspots of antivirals (such as tecovirimat, cidofovir, brincidofovir), vaccines (JYNNEOS, ACAM2000), herbal medicine and gene therapy.

**Supplementary Information:**

The online version contains supplementary material available at 10.1186/s12879-024-09252-w.

## Introduction

The current monkeypox outbreak was discovered in the UK on 7 May 2022. As of 9 May 2023, 111 countries and territories have been affected by the outbreak, with more than 87,314 confirmed cases of monkeypox and 129 deaths reported [1].

The monkeypox virus belongs to the Orthopoxvirus genus and causes a zoonotic disease similar to variola [2]. The monkeypox virus was first isolated from monkeys in 1958 by the Statens Serum Institute in Copenhagen, Denmark [3]. The virus can be accidentally transmitted to humans when they encounter infected animals [4]. In humans infected with monkeypox, the clinical symptoms are similar to those of variola, including fever, rash and swollen lymph nodes [5]. The monkeypox virus without targeted drugs is less infectious than the variola virus Most of the drugs used to treat monkeypox are antiviral drugs developed to prevent variola [6].

The multi-country outbreak of monkeypox led the World Health Organization (WHO) to declared monkeypox a Public Health Emergency of International Concern (PHEIC) [7]. A systematic description of monkeypox virus development trends and technical directions will help researchers around the world conduct targeted research; help enterprises identify opportunities for cooperation and avoid parallel activities, greatly improving the cooperative development of useful chemical compounds; and help policy-makers understand the virus, supporting efforts to prevent the spread of this disease. Innovation studies have shown that patent analysis is a very advantageous approach to describe global trends in technological knowledge production because it provide systematic information decomposed and attributed to extremely detailed geospatial and temporal technological domain hierarchies [8]. The World Intellectual Property Office (WIPO) has issued guidelines for the preparation of patent landscape reports to provide businesses, academia and government agencies with more standardized patent analysis results [9].

However, patent documents are often overlooked as useful sources of information. No patent landscaping reports have been published in relation to the monkeypox virus, combining temporal trends, geographic distribution, social networks and statistical methods. Patent mining and landscaping of the monkeypox virus is urgently needed. To fill in these gaps, we provide an overview of current trends in efforts to address the monkeypox virus—focusing on the patent landscape in time, space, economic dimensions, and technology dimension—and adopt a knowledge flow network perspective [10] and statistical methods to analyze patent relationships. The results of this study can provide a reference for enterprise investment, academic research and government decision-making.

## Materials and methods

The Derwent patent database (https://clarivate.com/products/derwent-innovation/) reflecting world patent information was used for this study to search a series of search terms related to “monkeypox” and to obtain 1,791 patent documents with priority dates before June 15, 2022. For a more comprehensive analysis of monkeypox virus, various data, such as inventors, patentees, international patent classification (IPC), citations, etc., were considered in this study. The patent analysis described below included time trends, geographic analysis, patentees and collaborators, patent transfer, technology categories, and citations.

This study employed network analysis to analyze the patterns and dynamics of the global monkeypox-related knowledge flow. The complex network analysis software Gephi was used to visualize monkeypox-related patent collaboration networks, patent transfer and citation networks. Linear regression model was used to study the influencing factors of patent citation times. Pajek was used to conduct main path analysis to explore milestone patents. Preferred reporting items for systematic reviews and meta-analyses (PRISMA) was used as the reference to simultaneously collect all patent documents worldwide and exclude any unrelated data. In addition, we also reported items according to the reporting items for patent landscapes (RIPL) checklist [11, 12]. The complete methodology employed in this study is described in the supplementary material.

## Results

### Data overview

**Fig. 1:**
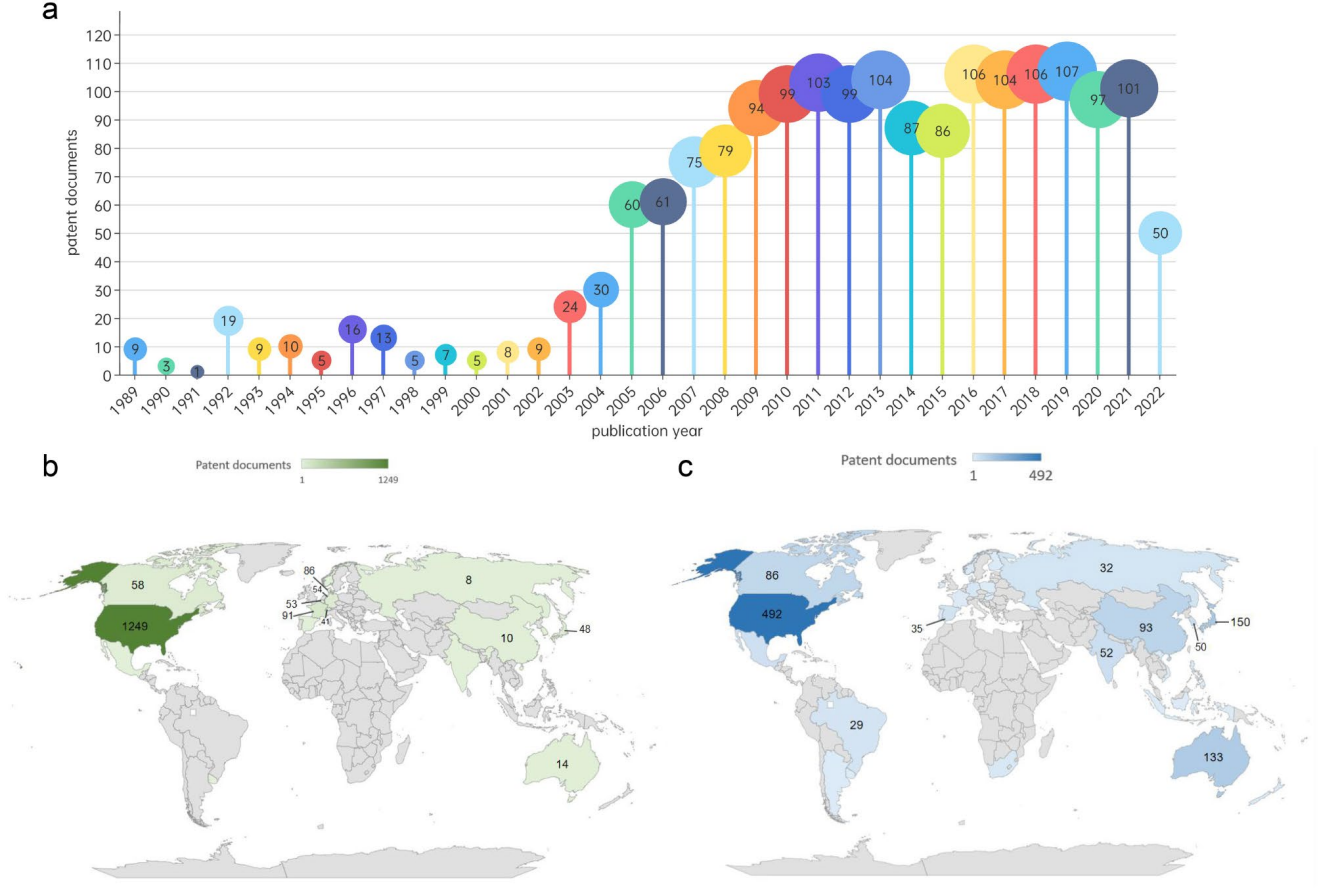
Temporal and geographic distribution of monkeypox virus patents. (**a**) Publication trend (based on patent documents). (**b**) Geographic distribution by nationalities of patent inventors. The color intensity denotes the frequency of patent documents. (**c**) Geographic distribution of patent disclosure. The color intensity denotes the frequency of patent documents

A dataset of 1,791 patent documents from 274 patent families was obtained. The supplementary information Fig.S1 shows the number of patents excluded and included at each stage and the related reasons. In 1989, the first monkeypox-related patent was filed, DK198901518 (monkeypox was first discovered by Danish virologist Preben von Magnus [13]), for treating monkeypox virus infection with dimethylol cyclobutylpurine and pyrimidine. Subsequently, monkeypox-related patents were published worldwide. In 2004, the number of monkeypox-related patents doubled and continued to rise annually. However, the number of published monkeypox-related patents declined significantly from 2014 to 2015 and did not return to a stable state until 2016 (Fig. 1.a).

### Geographical distribution

This study analyzes the technical origin and market destination of patents (patent inventor region and patent disclosure region) to explore the source and destination of patents, which is a common approach in innovation research. As shown in Fig. 1.b, most monkeypox-related patent inventors are in the US (1,249 patents). France is the second largest number of patent inventors, with 91 patents being created by people of French nationality. In third place, Danish inventors filed 89 patents. The top 6 countries in terms of patent inventors were analyzed (supplementary information Fig.S3). The “Two-Letter codes” by full country names are shown in supplementary information Table S4. Before the 21st century, patent inventors were concentrated in the US, and the number of invention patents fluctuated by approximately 8 per year on average. The number of inventors in the US has been on the rise since 2000, peaking in 2019 (77 patents). In 2001, inventors in France began to enter the field of monkeypox virus-related therapeutic innovations and apply for monkeypox-related patents. Denmark was the first country to disclose a patent for monkeypox, but inventors in Denmark began filing patents in 2003, well after 1989.

The patent market can be explored by patent disclosure areas and patent offices (Fig. 1.c), in which the US has published 492 patent documents, ranking first. Japan (150 patents), Australia (133 patents), China (93 patents), and Canada (86 patents) have published fewer patents than the US. The WIPO and the European Patent Office (EP) have published 203 patents and 167 patents, respectively, expanding the market reach of monkeypox-related technologies. The top 5 patent disclosure areas were analyzed (supplementary information Fig.S4). Before 2002, the number of patents disclosed in the patent disclosure areas was fewer than five per year. However, the total number of patents disclosed in the US has increased rapidly since 2002, which far exceeds the number of patents disclosed in other countries each year. Although France and Denmark are important countries in the origin of technology, they are not dominant in the market. In Japan and Australia, the number of patents published each year has shown a steady development trend, fluctuating in the range of 5 to 10 patents annually.

### Patentees and collaboration

**Fig. 2:**
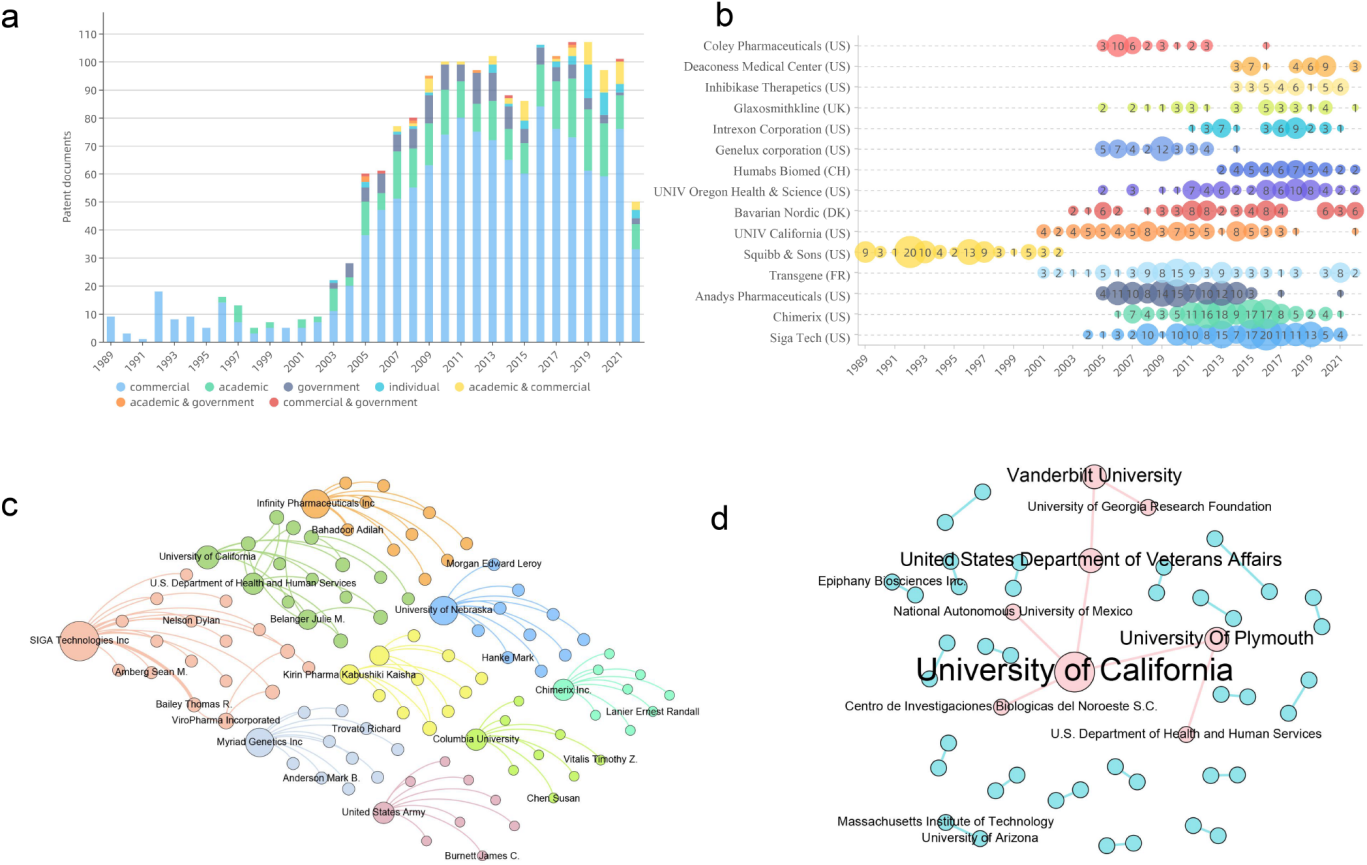
Temporal trends and cooperative network of patentees. (**a**) Publication trends of different patentees. (**b**) Temporal trends of patent applications by the top 15 patentees (based on patent documents). Different colors represent different institutions. (**c**) The patentee cooperative network that includes individuals. The nodes represent the patentees. The larger the node, the more patents were filed by patentees together. The weight of the sides represents the amount of cooperation between the patentees. (**d**) Institutional cooperation network. Nodes represent institutions, and edges represent the cooperation between institutions. Pink communities represent the collaboration between multiple patentees, and blue communities represent the collaboration between two patentees

Patentees of monkeypox-related patents were classified. Most patents are filed by commercial institutions (1,271 patents), indicating that enterprises are the main contributors to monkeypox-related innovation (Fig. 2.a). In 1996, academic institutions began to focus on monkeypox-related patents. Monkeypox-related patents have remained stable since 2004, with the second-highest total number of patents (283 patents) occurring in that year. Governments and individuals have not taken a great interest in pursuing monkeypox-related patents, with only 127 patents and 44 patents in total, respectively. In the category of patentees, cooperation between commercial institutions and academic institutions dominates (53 patents). However, this cooperation only accounts for 2.96% of the total number of patents, indicating that the monkeypox virus has received little attention in cross-domain cooperation.

We analyzed the top 15 patentees in terms of the number of patents (Fig. 2.b). The top five patentees are commercial institutions, led by SIGA Technologies who developing products to treat monkeypox. However, SIGA Technologies was not the first commercial institution to file monkeypox-related patents. Prior to 2002, monkeypox-related patents were mainly filed by Bristol Myers Squibb (including the first monkeypox-related patent, DK198901518). In 2002, the University of California began applying for monkeypox-related patents. However, the University of California stopped patenting monkeypox-related therapeutic innovations after 2018 and did not step back into the field (WO2022055998) until 2022. Organ Health & Science University began to apply for monkeypox-related patents in 2005, and the number of patents peaked in 2011, followed by a decline. By 2018, Organ Health & Science University had filed 10 patent filings.

To accurately depict the cooperation of patentees in monkeypox-related therapeutic innovations, we mapped the major collaboration relationships and pattern networks among patentees. SIGA Technologies (US), filing the most monkeypox-related patents, also cooperates with other organizations most frequently (Fig. 2.c). Collaborators with SIGA Technologies are mainly individuals, among whom Bailey Thomas R has applied for the most monkeypox-related patents along with SIGA Technologies. Also participating in collaborations are the University of California (US), Infinite Pharmaceuticals (US), Myriad Genetics (US), the University of Nebraska (US), and Columbia University (US). These findings indicate that among the top collaborators on monkeypox-related inventions, the US occupies the main position. When considering cooperation between institutions (not counting individuals), the University of California is the core institution of cooperation (Fig. 2.d). With the University of California at its core, members of the collaborative network include Plymouth University (England), the National Autonomous University of Mexico (Mexico), the US Department of Veterans Affairs (US), Vanderbilt University (US), etc., showing that the US not only has a large number of domestic partners but also vigorously develops international technical cooperation.

### Patent transfer

**Fig. 3:**
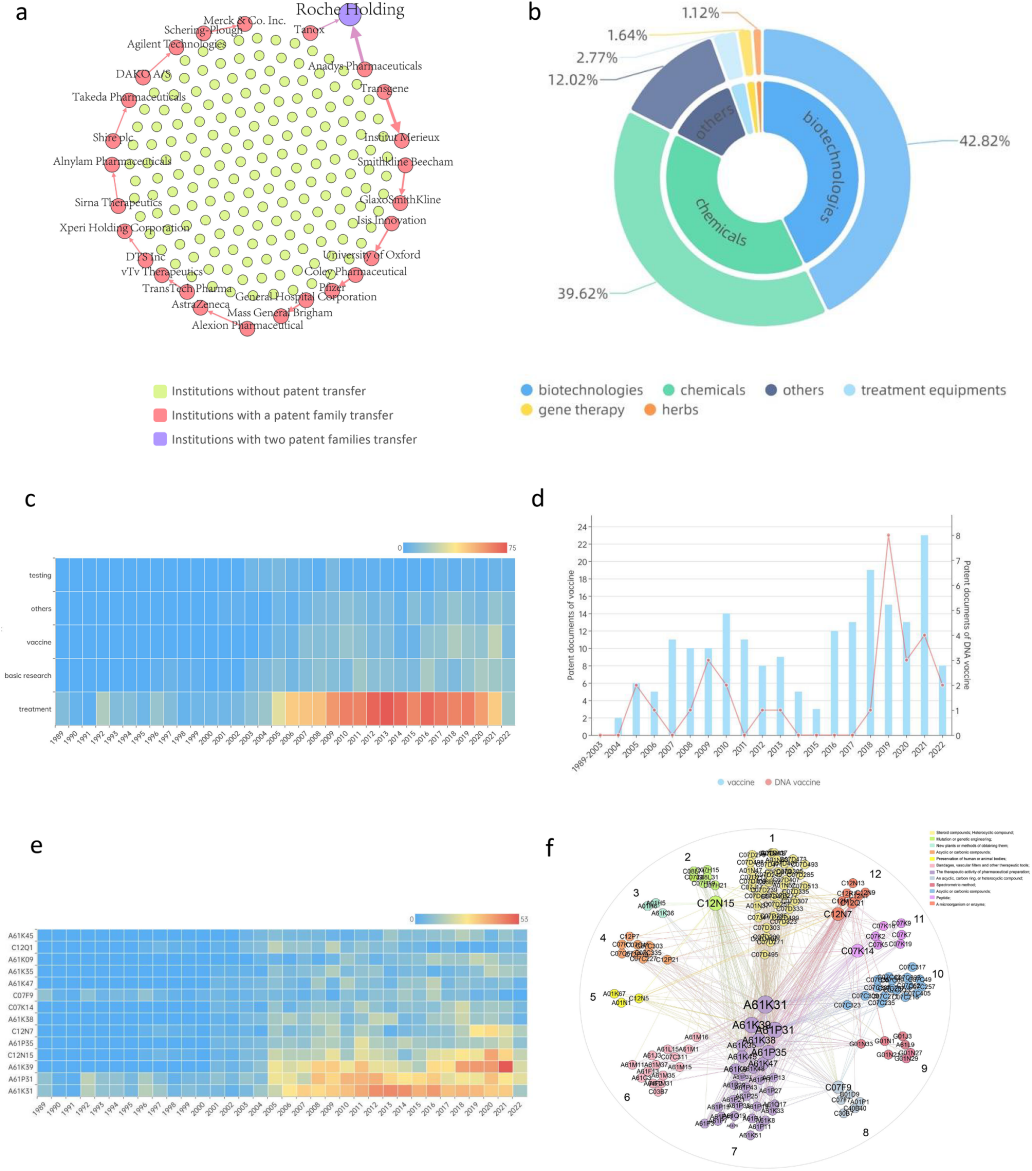
Network of patent license and analysis of technology. (**a**) Patent licensing network. Nodes represent institutions, and edges represent the direction of patent transfer. The node size represents the number of transferred patent families, and the weight of the edge represents the number of transferred patent documents. In the transfer network, the yellow communities are the institutions without patent transfers, the pink communities are where patents circulate between two institutions, and the purple communities are home to the largest number of patent families transferred to institutions. (**b**) Technological classification. Treatment areas are classified in detail, with patent documents gradually decreasing in a clockwise direction. (**c**) Time slice of technological trend. The trend of technology categories over time represents an increase in the number of patent documents from blue to red. (**d**) The development trend of monkeypox vaccines. (**e**) The temporal trend of IPC characteristics. From blue to red, represents an increase in the number of patent documents. (**f**) Technical characteristics network. Different-colored communities represent different technical characteristics, which are classified in detail under the “Technical characteristic network” in supplementary information Table S2

To explore the patent value of the monkeypox-related therapeutic innovations, the mode network and influencing factors of patent transfer were studied from the economic dimension, one of the four dimensions of patent analysis (technical dimension, legal dimension, economic dimension and talent dimension). As shown in Fig. 3.a, Roche Holding (Switzerland) has received the largest number of patents, with Anadys Pharmaceuticals (US) transferring 94 patent documents and Tanox (US) transferring one patent. Transgene (France) has transferred 83 patent documents to Institut Merieux (France), the second-highest number of patent transfers. SmithKline Beecham (US) has transferred 28 patent documents to GlaxoSmithKline (UK), the same number that Coley Pharmaceutical (US) has transferred to Pfizer (US). SIGA Technologies filed the largest number of patent documents without being involved in transfers to commercial or academic institutions.

### Technology

#### Technical types

The study classified patents into 5 technical types (supplementary information Fig.S2 and Table S3), with the treatments containing most patent documents (1,156 patent documents). Moreover, the number of patents on treatments has grown rapidly since 2001. Biological drugs ranked first (42.82%), followed by chemical drugs (39.62%) (Fig. 3.b).

However, monkeypox-related patents were present only in the therapeutic realm prior to 1995. In 1996, two basic research patents (WO1996011280 and AU199538327) appeared, disclosing the non-naturally occurring attenuated virus. The attenuated virus can enhance the ability of the human body to fight monkeypox virus. In 2003, three patents of detecting monkeypox virus were published (WO2003066807, US20030211964 and WO2003046221), describing the identification of monkeypox virus by DNA-DNA and Z-DNA. In 2004, patents for recombinant poxvirus vaccines to prevent monkeypox infection were published (WO2004005523 and AU2003281290) (Fig. 3.c). According to research on vaccines, DNA vaccines only account for 14.72% of the total number of monkeypox vaccine-related patents, more stable and easier to store and transport [14]. In the past five years, DNA vaccine-related patents have been published with the improvement of vaccine production technology. The number of DNA vaccine patents published in 2019 accounted for 53.33% of the total number of vaccine patents that year (Fig. 3.d).

#### IPC characteristics

To depict the changes in the technology direction and investment trends of patent applications, we calculated the temporal trend of IPC (top 13 in the number of patent documents). The IPC categories of patent indicate the technical field of invention, and the most involved technical field is A61K31 (pharmaceutical preparations containing organic active ingredients). In 2005, A61P31 (anti-infective drug), A61K39 (remedy containing antigen or antibody), and C12N15 (mutation or genetic engineering) increased significantly and fluctuated mildly in the following years. As antibody drugs gradually became a potential stock in the development of the biomedical field, A61K39 (53 patents) reached the peak in 2021. C12N7 (composition and preparation of viruses), C07K14 (peptide with more than 20 amino acids), A61K35 (medical preparations), and C12Q1 (containing nucleic acid or microbial determination methods) also showed significant increases in 2005 but did not continue to increase thereafter. In 2011, A61P35 and C07K14 suddenly increased. In 2019, patents for C12N7 and A61K9 (pharmaceutical compounds) increased significantly (Fig. 3.e).

Using social network analysis to further explore the monkeypox-related technical research direction, we classified IPC into 12 communities (classification criteria in supplementary information Table S2). There are 152 nodes (IPC) and 664 undirected edges (patents assigned to multiple IPC categories). Community 1 (yellow) has the highest number of IPC members, which are biocides, steroid compounds, and heterocyclic compounds. Community 7 has the largest number of patents related to A61K31, A61P31, A61K39, A61K38 and other pharmaceutical products (Fig. 3.f). Overall, the monkeypox-related patents account for the largest proportion of pharmaceutical formulations, also corresponding to the first monkeypox-related patent, which indicates that in the field of the monkeypox virus, enterprises and academic circles focus on the research and development (R&D) of drugs for treatment.

#### Patent citation and milestone patents

**Fig. 4:**
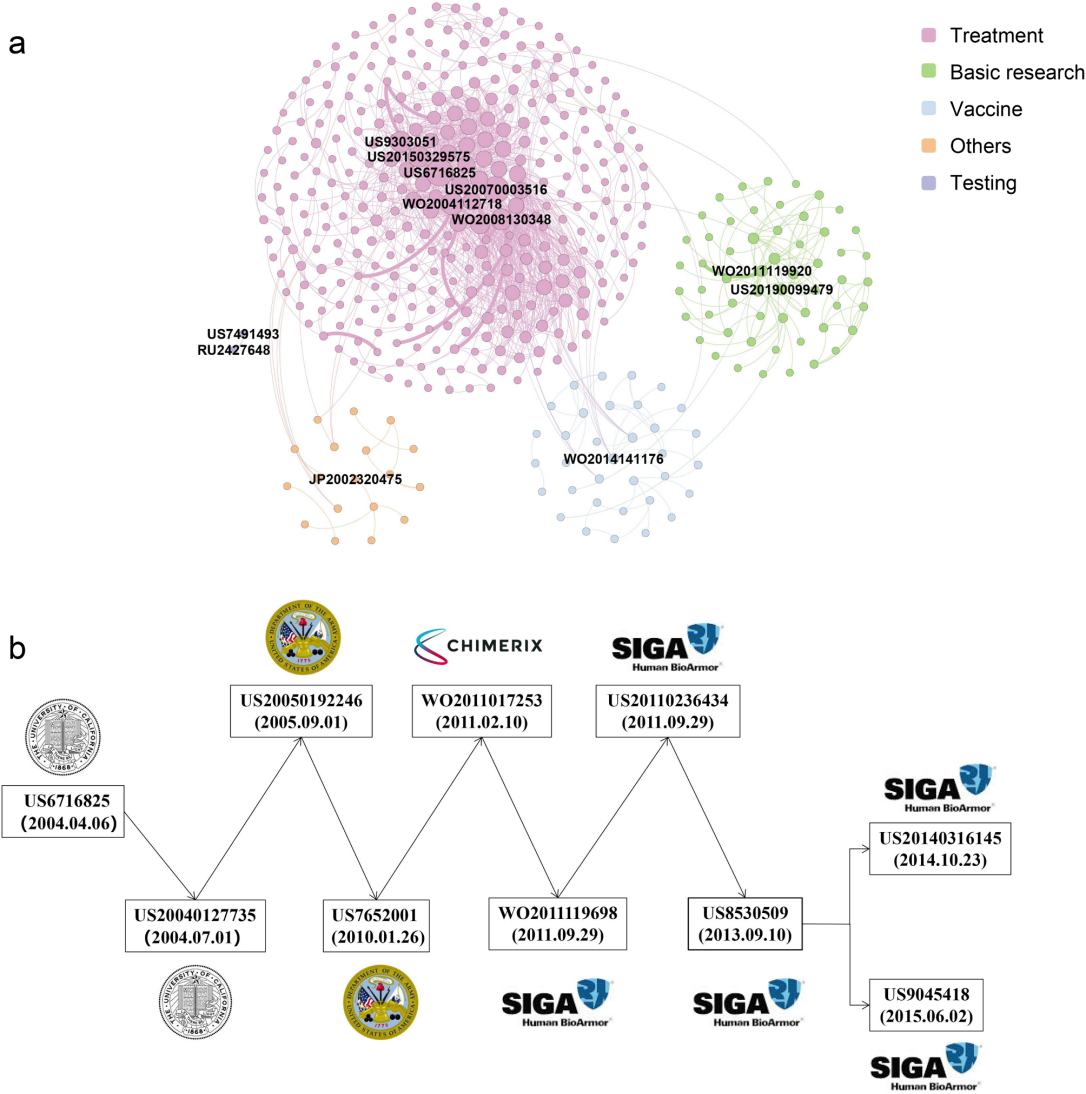
Citation network and main path analysis of monkeypox virus. (a) Global citation network. Including all patent documents and their citation links. Larger nodes represent patents that have been cited more often. The node size is set according to its output value. The larger the output value is, the larger the node size and the more times a given patent has been cited. To separate the different technologies, we divided the technology categories into five communities, as described in supplementary information Table S1 and Fig.S2. Then, the layout was presented through the technical classification of nodes (patents), and the “grid layout” strategy of Gephi software was used to reference the network in the same technical module. (b) Main path. Pajek was used for main path analysis of patent citation and landmark patents were labeled

From the technical dimension, we explored the milestone patents related to the monkeypox virus. Figure 4.a shows the patent citation network with 437 nodes and 1,201 edges. Different-colored communities represent different technology types. The more times a patent is cited, the larger the node, which means that the patent has greater impacts on subsequent inventions. The pink community has the largest number of nodes (74.6%), consistent with the analysis results of technology categories and technology characteristics. Most of the frequently cited patents are in the pink cluster, including the most frequently cited patent (US6716825) describing the use of phosphonate compounds by the University of California to treat monkeypox infections. However, only two patents are cited for the purple community (0.46%), indicating that scientists are still unfamiliar with the field of monkeypox virus detection and lack technical support.

Unary linear analysis and multivariate linear analysis were used to determine predictors of patent citations, and 95% confidence intervals (*p* < 0.05) were used to indicate the statistical significance of the results. All statistical analyses were conducted using statistical package for the social sciences (SPSS) software version 25.0 (SPSS Inc.). Table 1 shows all the predictors of monkeypox patent citations. Year of publication (OR: 0.451; 0.167 ∼ 0.734), Non-patent citations (OR: 0.382; 0.340 ∼ 0.424), Citation literature (OR: 0.511; 0.012 ∼ 0.230), Jurisdiction district of WO (OR: -10.022; -15.624∼-4.420), Jurisdiction district of Japan (OR: -13.045; -19.203∼-6.888), Jurisdiction district of Australia (OR:-11.743; -18.041∼-5.446), Jurisdiction district of China (OR: -11.426; -18.577 ∼ -4.276), Jurisdiction district of Canada (OR: -12.462; -19.835 ∼ -5.088), and Jurisdiction district of other areas (OR: -12.144; -16.842 ∼ -7.446) are significant influencing factors of monkeypox-related patent citations. For further details, please refer to supplementary information Table 1.

**Table 1:** Linear regression analysis to identify influencing factor of patent citation

Related categories	Unary linear analysis			Multivariate linear analysis		
	Odds Ratio for citation	95% CI	P-value	Odds Ratio for citation	95% CI	P-value
**Year of publication**	0.420	( 0. 174 ∼ 0.666 )	0.001***	0.451	( 0. 167 ∼ 0.734 )	0.002**
**Inventors**	0.500	(−0.358 ∼ 1.358 )	0.253	−0.416	(− 1.202 ∼ 0.370 )	0.299
**Claims**	−0. 101	( −0.262 ∼ 0.059 )	0.216	0.045	( −0. 100 ∼ 0. 191 )	0.539
**IPC**	0. 143	( −0.070 ∼ 0.355 )	0. 188	0.238	( 0.020 ∼ 0.456 )	0.032*
**Non-patent citations**	0.450	( 0.411 ∼ 0.489 )	0.000***	0.382	( 0.340 ∼ 0.424 )	0.000***
**Citation literature**	0.403	( 0.294 ∼ 0.511 )	0.000***	0.511	( 0.012 ∼ 0.230 )	0.029*
**Applicant**	1.293	( −0.097 ∼ 2.682 )	0.068	0.316	(− 1.061 ∼ 1.693 )	0.653
**Type of patentees**						
**Commercial (Reference category)**	1.000			1.000		
**Academic**	0.639	( −3.974 ∼ 5.251 )	0.786	0. 166	( −4. 180 ∼ 4.512 )	0.940
**Government**	1.992	( −4.577 ∼ 8.561 )	0.552	−3.656	( −9.995 ∼ 2.683 )	0.258
**Academic** **&Commercial**	−6.511	(−16.336 ∼ 3.315 )	0. 194	−7.266	(− 16.046 ∼ 1.513 )	0. 105
**Individual**	−6.766	(−17.513 ∼ 3.981 )	0.217	−2.430	(− 12.622 ∼ 7.762 )	0.640
**Academic** **&Government**	−6.009	(−32.573 ∼ 20.554 )	0.657	− 1.505	(−25.008 ∼ 21.998 )	0.900
**Government** **&Commercial**	−6.486	(−35.166 ∼ 22.195 )	0.657	− 1.399	(−26.590 ∼ 23.792 )	0.913
**Countries of patentees**						
**US** **(Reference category)**	1.000			1.000		
**FR**	−8.378	(−15.961 ∼ −0.795 )	0.030*	−4. 182	(− 11.087 ∼ 2.722 )	0.235
**DK**	−2.234	(− 10.020 ∼ 5.552 )	0.574	−2.865	(− 11.435 ∼ 5.706 )	0.512
**GB**	−10.557	(−20.179 ∼ −0.935 )	0.032*	−2. 155	(− 10.874 ∼ 7. 168 )	0.650
**CA**	−7.445	(− 16.825 ∼ 1.936 )	0. 120	− 1.832	(− 11.479 ∼ 7.210 )	0.691
**Other countries**	−6.838	(− 11.660 ∼ −2.015 )	0.005**	−3.426	( −7.968 ∼ 1. 116 )	0. 139
**Technological types**						
**Treatment (Reference category)**	1.000			1.000		
**Other technologies**	−5.537	(− 11.700 ∼ 0.626 )	0.078	−2.061	( −7.635 ∼ 3.513 )	0.468
**Testing**	−9.612	(-18.949 ∼ −0.275 )	0.044*	−2.948	(− 11.663 ∼ 5.767 )	0.507
**Vaccine**	−5.004	( −10.419 ∼ 0.412 )	0.070	−3.226	( −8.791 ∼ 2.340 )	0.256
**Basic research**	−2. 140	( −7. 145 ∼ 2.866 )	0.402	0.666	( −4.832 ∼ 6. 163 )	0.812
**Within the validity period of patent protection**						
**No** **(Reference category)**	1.000			1.000		
**Yes**	7.849	(−4.382 ∼ 11.316 )	0.000***	3. 109	( −0.650 ∼ 6.868 )	0. 105
**Indeterminate**	−4.801	( −10.578 ∼ 0.976 )	0. 103	−3.696	( −9.703 ∼ 2.310 )	0.228
**Jurisdiction district**						
**US** **(Reference category)**	1.000			1.000		
**WO**	− 18.389	(−23.983∼−12.794 )	0.000***	− 10.022	(− 15.624 ∼ −4.420 )	0.000***
**EP**	− 12.777	( −18.783 ∼−6.771 )	0.000***	−3.516	(− 9.325 ∼ 2.294 )	0.235
**JP**	−23. 178	(−29.433 ∼−16.923 )	0.000***	− 13.045	(− 19.203 ∼ −6.888 )	0.000***
**AU**	−24.825	(−31.379 ∼ −18.270 )	0.000***	− 11.743	(− 18.041 ∼ −5.446 )	0.000***
**CN**	−24. 182	(−31.765 ∼ − 16.599 )	0.000***	− 11.426	(− 18.577 ∼ −4.276 )	0.002**
**CA**	−26. 118	(−33.956 ∼ − 18.280 )	0.000***	− 12.462	(− 19.835 ∼ −5.088 )	0.001***
**IN**	−26. 118	(−35.897 ∼ − 16.339 )	0.000***	− 10.729	( −20.015 ∼ − 1.442 )	0.024*
**KR**	−24.298	(−34.252 ∼ − 14.343 )	0.000***	− 11. 109	( −20.471 ∼ − 1.747 )	0.020*
**Other areas**	−25. 140	(−29.773 ∼ −20.507 )	0.000***	− 12. 144	(− 16.842 ∼ −7.446 )	0.000***

*Notes* p* < 0.05; p** ≤ 0.01; p*** ≤ 0.001

Main path analysis was conducted to uncover landmark patents. Figure 4.b shows that US6716825 is the core patent, consistent with the results of the patent citation network analysis. The next patent (US20040127735) published in the same year is from the same family as patent US6716825. US20050192246 and US7652001 were applications by the US as represented by the Secretary of the Army for the use of alkyl esters of phosphonates for treating viral infections. WO2011017253 was an application by Chimerix for the use of phosphonic acid derivatives for treating viral infections. WO2011119698, US20110236434, US8530509, US20140316145 and US9045418 were all drugs applied by SIGA Technology for treating Orthopoxvirus infection.

## Discussion

In this paper, we review the monkeypox-related patents, generating patent landscapes and mining. This study determines the global pattern of monkeypox virus-related patents from 1989, when the first patent was published, to 2022, including the temporal trend, geographical distribution, partnership of patentees, patent transfer, influencing factors of patent citations and key points of patented technology. The temporal trend shows that attention to the monkeypox virus has increased significantly since the 21st century, and patent activity has followed the rapidly increasing trend of the monkeypox virus epidemic.

### Several outbreaks of monkeypox

Human infection with monkeypox was first identified in 1970 in the Democratic Republic of Congo [15, 16]. Since then, most human cases have occurred in the rainforest regions of West and Central Africa for reasons likely linked to factors of deforestation and climate change, increasing the degree of close contact between humans and animals [17]. Civil wars in African countries may force people to seek new sources of food, such as monkeys and squirrels [18]. However, the first monkeypox-related patent was not filed until 1989 (DK198901518), 19 years after the first human infection. In 1996 and 1997, Congo reported the largest outbreak of monkeypox ever recorded [19]. Those two years saw a significant rise in the number of monkeypox patents (16 and 13, respectively). In 2003, more than 70 cases of monkeypox appeared in the US, the first human case outside Africa, drawing the attention of American agencies [20]. Although the monkeypox epidemic is concentrated in Africa with weak resources for surveillance, diagnosis, and even patient care, our analysis of patent temporal trends and geography shows that the US dominates the monkeypox-related patent trend, with far more published patent documents (492) and patent inventors (1,249) than any other country. After this outbreak, the number of monkeypox infections dropped. In 2017, there was an outbreak of monkeypox in Nigeria [21], causing the monkeypox virus to gradually spread to other countries. In May 2022, multiple cases were identified in the UK, confirming an ongoing outbreak of the monkeypox virus [22].

### Geographical biases

The worldwide monkeypox patent landscape shows strong geographical biases. In view of the patent inventor and disclosure regions in Fig. 1.b and Fig. 1.c, developed countries are innovation cradles and are at the center of a growing influx of patented inventions, in contrast with the developing regions. The issue of technology transfer from developed countries to developing ones is a barrier to the sustainable application of developed technology [23]. To solve this problem, government incentives may play important roles, especially in developing countries. The national governments are encouraged to identify and fund the research and technology essential to fighting monkeypox-related production. Government subsidies, promotional campaigns, and monkeypox techniques mandated in national policies can be implemented to facilitate the development of first-generation vaccines and medications [24]. Direct financial support, such as research granted by tailoring government contracts of patent licensing used to fund medical research. Licensing provisions that obligate recipients of government funding to share relevant technology can overcome obstacles that intellectual property rights (IP) present [25].

As the most dominant country in patent licensing, the US holds the most influential patents, is linked to other countries most widely, and has the widest range of cooperation partners (Fig. 1.b, Fig. 1.c, Fig. 2.b, Fig. 2.d and Fig. 4.b). Other than the advanced R&D environment, it may be due to the fact that the US extremely benefits from the extensive mode of academia-industry collaboration. But without the transparent commercial licensing practices, there may be the potential formation of a patent thicket, and this laissez-faire approach may take obstructive practices for global health [26].

On the one hand, as a field matures, industry partnerships and buyouts typically consolidate patents [26]. The collaborative efforts in patent support should be provided for small biotech companies worldwide, in regard to the merger and acquisition (M&A) of Anadys Pharmaceuticals (US) by Roche Holding (Switzerland). On the other hand, an open innovation model highlighting the multisector and multinational collaborative culture in partnerships should be well established [27]. The stakeholders should equally embrace collaboration beyond the data sharing around patents to avoid damaging geographic barriers. For example, there are strategies like collaborative approach through transparent commercial licensing practices in online platforms, nonconfidential trading activity for smaller biotech collaboration with universities, and creating a global IP registry and exchange market.

In addition, it is encouraged to make low licensing fees for trading partners from developing regions. Government and not-for-profit institutions should develop the patent transfer agreements, which prohibit the unreasonable access fees [28]. These actions would go far to remove the perceived threat of geographic barriers and better serve public health. All of the aforementioned challenges need to be considered and extensively researched in order to fight against monkeypox worldwide.

### Treatment, prevention and diagnosis

#### Treatment

In the treatment of monkeypox virus, the number of biological drugs and chemical drugs ranked at the top, and gene therapy and herbal therapy only accounted for 1.64% and 1.21%, respectively. Gene therapy, as a new technology combining modern medicine and molecular biology, has made some achievements in treating diseases. In particular, the University of California filed a patent (WO2022055998) in 2022 proposing the use of CRISPR gene therapy to treat monkeypox virus infection, which has become one of the powerful strategies for treating monkeypox virus in the current gene therapy trend. Traditional herbs are also potential treatments for immunodeficient patients infected with monkeypox virus. Patents reveal the preparation method of pharmaceutical compositions to treat monkeypox infection from Croton plants (US20060204600), medicinal mushrooms (US20110008384), bilberries (WO1997041137), and other plants (WO2014081976, US20120302733). However, there is no specific drug targeting the monkeypox virus [29]. The only current treatment is symptomatic treatment. In response, doctors are now using drugs developed for treating variola to treat monkeypox. The approach of drug repurposing provides cheap, effective and safe monkeypox drugs with few side effects and speeds up the monkeypox drug development process [30].

Tecovirimat, an antiviral drug (WO2015077143) developed by SIGA Technologies, is considered to be the first-choice treatment for the variola virus [31]. Tecovirimat has been shown to inhibit the replication of various orthopoxviruses in cell cultures in vitro [32], including monkeypox virus [33]. Patents (US7687641, CA2866037, WO2011119698) demonstrate that SIGA Technologies has mastered the process and formula of tecovirimat, and constantly updated the preparation method of tecovirimat, becoming the enterprise with the most patent applications. However, when the monkeypox outbreak hit in 2022, the Food and Drug Administration (FDA) had not approved tecovirimat for treating monkeypox, and stressed that clinical trials were needed to demonstrate the safety and effectiveness of tecovirimat for monkeypox treatment. The move has raised the alarm of some infectious scientists, who have criticized FDA regulatory barriers for making tecovirimat less accessible to patients at a time when monkeypox cases are soaring in the US. These scientists want the FDA to issue an Emergency Use Authorization (EUA) for tecovirimat [34, 35].

Chimerix, the company with the second largest number of monkeypox patent filings, is offering a variola antiviral drug brincidofovir for all age groups [36], which is an analogue of the intravenous drug cidofovir with a higher safety profile. Chimerix offers a high purity production method for cidofovir (JP2017214378), and mentions to use cidofovir in combination with tecovirimat or vaccinia immune globulin to treat monkeypox virus infection. University of California, Brigham Young University, Merck & Co. have also filed cidofovir-related patents (WO2001038724, US20070190066). And they conducted studies to detect antiviral activity of drugs, improve possible adverse reactions after vaccination, and inhibit symptoms of poxvirus infection after using vaccines (such as JYNNEOS, ACAM2000) or antiviral drugs (cidofovir). In June 2021, the FDA approved brincidofovir to treat patients with variola, and it has been shown to be effective against monkeypox in vitro and in animal studies [37].

#### Prevention

For monkeypox vaccine research, there is a lack of patent support (Fig. 3.c and supplementary information Table S3). There is currently no vaccine for the monkeypox virus on the market and the variola vaccine is considered to be 85% effective against the infection of monkeypox virus [38]. JYNNEOS, a variola vaccine for preventing the infection of monkeypox, was developed by Bavarian Nordic, a Danish company that has contributed to monkeypox virus research, ranking seventh in monkeypox-related patent applications [39–41]. ACAM2000 is a attenuated, replicable vaccinia virus vaccine first licensed against monkeypox in 2015 [42]. However, JYNNEOS is only suitable for prevention of monkeypox in susceptible adults aged 18 years or older, and serious side effects after inoculation with vaccinia virus similar to ACAM2000 exist. According to the patents (CN112543806, WO2018085582), institutes are developing synthetic chimeric vaccinia viruses based on the modified Ankara-Bavarian Nordic vaccinia vaccine (JYNNEOS from the modified Ankara-Bavarian Nordic vaccinia vaccine) and the genome of ACAM2000, which can be used as a live viral vaccine against monkeypox virus. Currently, Bavarian Nordic holds IP to JYNNEOS, protecting its exclusive ownership of the development and production of the vaccine. However, the protective effects of IP can slow the process of vaccine production and distribution, with no end in sight to its limitations, putting lives at risk by depriving some parts of the world of resources for treatment and prevention. For example, there is still a shortage of vaccines that can prevent the infection of monkeypox virus in Africa, as Bavarian Nordic maintains control over vaccines [43, 44]. The vast majority of monkeypox vaccines are currently purchased in the US, with some going to other high-income countries. In addition, the R&D of vaccines is a complex and long-term process. In order to identify the effectiveness and safety of vaccines, clinical trials and strict approval pathways under supervision are mandatory, which will slow down the filing of vaccine patents [45]. Furthermore, it is also reported that only a few smallpox vaccine patents now applied for monkeypox had a patent portfolio and one under patent litigation processes in three continents because of potential IP barrier [46].

Therefore, when dealing with global infectious diseases like monkeypox, international multilateral models of cooperation should be encouraged to accelerate the R&D and distribution of vaccines, and to improve production techniques. Meanwhile, the adoption of open-source R&D models, EUA and other measures are encouraged, which make patent protection no longer the primary consideration.

#### Diagnosis

We have shown that the techniques for detecting Monkeypox virus mainly include polymerase chain reaction (PCR) (WO2004092420), high-throughput method (WO2005123966, CA2570296) and isothermal amplification detection (CA2570296), which are the basis for large-scale detection of human populations and the effective ways to prevent the outbreak of monkeypox [47]. However, large-scale detection of human populations requires countries to have adequate infrastructure and robust health systems, which is one of the major challenges in the global fight against monkeypox outbreaks. Despite the WHO suggesting that supporting low-income and middle-income countries to have access to monkeypox diagnostics is a priority, there are barriers to implementing molecular diagnostics for monkeypox in those countries along with chronically weaker health systems, lacking of development of rapid tests [48]. In addition, patent activity is often driven by market demand and scientific research directions. Since the R&D of drugs has always been a more focused area and a major investment target for pharmaceutical companies, the potential growth of market profitability of treatment tends to be greater than virus testing, bringing relatively little patent activity related to detection innovation compared with medication. There is an R&D need for early screening tools of virus before the next outbreak.

To sum up, when there is no major active outbreak, the need to have vaccine candidates and diagnosis instruments prepared before the onsets of such outbreaks necessitate targeted investments by commercial institutions, public and philanthropic institutions. Patent landscapes can be used by policymakers to identify potential solutions for those areas and redirect funding for further development of promising patent applications. It is recommended to focus funding resources on the cluster of patents that are classified in these technological areas of major applicants.

### Commercial perspective

Since the 21st century, the number of monkeypox-related patents has increased significantly, especially the number of commercial patents, which has shown explosive growth. This study demonstrates that monkeypox virus technology development is an issue of industrial and commercial importance.

Among the previous monkeypox patentees are SIGA Technologies and Chimerix, pharmaceutical companies founded in 1995 and 2000, respectively, which focus on the development of antiviral drugs for the variola virus and have strong R&D capability related to the monkeypox virus [49]. However, our main path analysis revealed that a landmark patent (US6716825) was obtained for the use of phosphonate compounds in the treatment of various medical diseases, including monkeypox infection, by the University of California. This finding indicates that although the R&D of monkeypox-related therapeutic innovations in enterprises is almost always used to explore commercial value, academic research on monkeypox has the greatest impact on subsequent inventions. There are many important problems in the industry, but there is not enough available labor to solve them, and only the most profitable problems can be chosen. Academics can focus on small but critical problems and develop breakthrough core technologies. Academic inventors are less willing to patent globally to gain market share under global patent protection.

In addition, the cooperation mode also reflects the difference between business and academia. The reason why the two networks with partners (SIGA Technologies and University of California) are inconsistent is that SIGA Technologies has a large number of collaborating R&D scientists, some of whom are hospital doctors but not employees of SIGA Technologies, such as Bailey Thomas R. These scientists are pioneers of SIGA Technologies. The drug tecovirimat, developed in collaboration with SIGA Technologies, can treat Orthopoxvirus infections [50]. The University of California, as the top university of immunology and infectious diseases in the US, occupies the core position of institutional cooperation in the research of monkeypox infectious diseases. Its closest partner, Plymouth University, has its own biomedical group specializing in viral infections, immunity and inflammation.

The reasons for monkeypox patent transfer were analyzed from a commercial perspective. Anadys Pharmaceuticals, which was acquired by Swiss drug maker Roche Holding in 2011 [51], invented antiviral drugs that stimulate a patient’s immune system and block infected cells from carrying the virus. Transgene (France), which is part of Institut Merieux (France) [52], transferred 83 patents to Institut Merieux, the second largest transfer of patents, and is dedicated to the development of therapeutic vaccines and immunotherapy products. The M&A and VIC (venture capital, intellectual property and contract research organization) models, which are generated by changes in the affiliation relationships between institutions, can save considerable capital investment. These models are the most efficient and low-cost innovative drug R&D at present and improve the innovation ability and market competitiveness of enterprises in subsequent R&D [53, 54].

Overall, monkeypox may not be commercially valuable to companies before a monkeypox epidemic, but pharmaceutical research into monkeypox is essential. The main process for enterprises to overcome the monkeypox virus is to conduct R&D on monkeypox, record the technology obtained from the research in patents, and convert these patents into marketable products to make profits. Therefore, when monkeypox virus does not pose a strong threat, enterprises should put more emphasis on monkeypox-related innovation, improve research efficiency and overcome the technology gap, so as to be prepared. For policy-makers, in addition to establishing an accelerated monkeypox patent review system, there is a need to accelerate issue emergency authorizations for medication use, reducing the barriers to the flow of innovative technologies and the inequalities in the areas to which these technologies are distributed [55].

### Limitations

However, this study also has some limitations regarding the database. Firstly, there is usually a time lag of at least 18 months between the first patent filing and the patent publication, and even longer time is used for granting [56]. Therefore, continuous tracking of monkeypox virus-related inventions in the coming years remains an effective way to tap into and validate emerging technologies. Secondly, the Derwent patent database containing 96% of the world’s patent information, is based on abstracts and claims of the English language, and has limited access to a few non-English full patents [57, 58]. To address this limitation, we recommend future research to make cross-validation with multiple sources, and to use other patent databases and academic publications as valuable supplemental data sources when deemed necessary, which would be more scientific, objective and reasonable. However, these are the general limitations of all patent landscape analyses.

## Conclusion

The number of monkeypox virus patents increases with outbreaks, especially in the US. Therapeutic innovations of monkeypox are geographically limited. In Africa, where outbreaks are common, far fewer monkeypox patents are filed and published than in the Americas because of weak resources for surveillance, diagnosis and even patient care. The US has the largest number of published patents and patent inventors. American companies and universities, including SIGA Technologies and Chimerix, dominate the patent cooperation network. The academic community of the University of California is an important technological force for global collaboration. The government’s contribution to monkeypox-related patents is modest, but it cannot be ignored in relation to national policy relating to the monkeypox virus. In the patent transfer network, the transfer of patent knowledge is facilitated by the M&A model and VIC model resulting from changes in the affiliation relationships between institutions. Antiviral drugs and antibody drugs have attracted much attention, but studies of vaccine and virus testing lack sufficient patent support. Patent protection is an ongoing process under commercial competition, especially in the global patent portfolio strategies of large enterprises. During pandemics, the role of patent rights is different from that under normal circumstances, and more attention should be given to the balance between the competition of interests and the general trend of a humanistic spirit and knowledge sharing.

### Electronic supplementary material

Below is the link to the electronic supplementary material.

Supplementary Material 1

## Data Availability

The data that support the findings of this study are available from the corresponding author Kunmeng Liu on reasonable request.

## Abbreviations

WHO: World health organization
PHEIC: Public health emergency of international concern
WIPO: World intellectual property office
IPC: International patent classification
PRISMA: Preferred reporting items for systematic reviews and meta-analyses
RIPL: Reporting items for patent landscapes
EP: European patent office
R&D: Research and development
SPSS: Statistical package for the social sciences
PCR: Polymerase chain reaction
EUA: Emergency use authorization
M&A: Merger and acquisition
VIC: Venture capital, intellectual property and contract research organization
IP: Intellectual property right
FDA: Food and drug administration
