# Supplementary material for: The global patent landscape of emerging infectious disease monkeypox
Source: BMC Infect Dis. 2024 Apr 15;24:403. doi: 10.1186/s12879-024-09252-w (PMC11017537; doi:10.1186/s12879-024-09252-w)
Supplement: Supplementary file 1 — Supplementary Material 1 [file 12879_2024_9252_MOESM1_ESM.docx]

The global patent landscape of emerging infectious disease monkeypox

1. **Supplementary method**
   1. **Dataset preparation**

The first step towards the preparation of patent landscaping and mining was taken by the construction of a dataset. This study retrieved patent documents from the Derwent Innovation® (Clarivate Analytics®) platform to obtain patent data sets for monkeypox virus related inventions. Keyword searches were performed in the title, abstract and claims sections of patent documents including the DWPI fields (Derwent World Patents Index®), which is expertly indexed and features enhanced titles and comprehensive abstracts. Keywords included the full names of the monkeypox, abbreviations, or synonyms. Search queries were executed based on the keyword: CTB=("monkeypox" or "monkey pox" or "MPX" or "MPXV" or "variole simienne" or "viruela del mono" or "Affenpocken" or "Affenpockenkrankheit" or "Affenpockenvirus" or "varíola dos macacos") OR TID=("monkeypox" or "monkey pox" or "MPX" or "MPXV") OR ABD=("monkeypox" or "monkey pox" or "MPX" or "MPXV"). The search was performed on June 15, 2022. Then we got 6,478 patent documents.

Besides we excluded irrelevant patents by double-checking manually, and then deduplicated records, e.g., different document types (e.g. A1, B2, C) of a given publication to avoid multiple counts for the same invention. As for the dataset cleaning, a fundamental data normalization step was performed to clean duplicate records that had occurred due to abbreviations or misspellings. The unrelated patents were deleted in the document records after being double-checked manually. Two authors (Cai and Zhang) worked together to select and assess patents, and they discussed any disagreement with the senior author (Liu). Here, after reading the title, abstract, claim and full text of patent files, we deleted all the patents unrelated to the monkeypox. Finally, we got 1791 patent documents (274 families) (Fig.S1). Moreover, after judging the relevance of classification to patent files by a hierarchical reading order from title, abstract, claims and full text, we added classification labels to relevant patent records.

- 1. **Classification criteria for patentees**

According to the nature of patentee, the patentees were divided into seven categories, namely commercial, academical, government, individual, academic and commercial, commercial and government, academical and government. Among them, the research institutes were classified as academic, and the hospitals as commercial because of its profitable nature.

1. **Supplementary table**

**Table S1.** The standardization of patent classification.

| Case | Classification |
| --- | --- |
| Title | If the classification information is clearly shown in the title of a patent document, we labelled the patent by the classification. For example, patent WO2013036993 is entitled “Detection of viral infection”. We chose the “testing” as the classification. |
| Abstract | If there is no classification information mentioned in the title, we further captured the classification information in the abstract. For example, the abstract of patent WO1997041137 is “The use of an anthocyanidin or its derivative for the preparation of a pharmaceutical composition for the prevention and/or treatment of neoplastic disorders and diseases caused by lesions in connective tissues or by viruses is new. The virus to be treated is selected from parvorira, such as monkeypox, smallpox and cowpox virus.” We chose the “treatment of herbs” as the classification. |
| Claims | If there is no classification information both in title and in abstract, we continued to check patent claims. In most cases, by reading the title and abstract, we can divide the patents into general categories. If we can't determine the subcategory by reading the title and summary, we need to refer to patents claims to classify the subcategories under the general category. Make decisions based on the level of priority claim. For example, patent WO2007034166 claims that “The vaccine compositions produce fewer side effects than currently available smallpox vaccines using live vaccinia viruses, and in particular are more suitable than current vaccines for treating immunocompromised individuals, such as individuals infected with HIV. The orthopoxvirus antigen is from monkeypox virus, cowpox virus, variola virus and so on.” We recognized that the patent covers “vaccine” as the classification. |
| Full-text | When none of the above ways can determine the category, we will read the full text. |

**Table S2.** Technical characteristic network.

| **community** | **IPC** | **Meaning of IPC** |
| --- | --- | --- |
| 1 | A01N47, A01N37, A01N43, A01N63, C07J41, C07J17, C07J9, C07D405, C07D471, C07D473, C07D513, C07D401, C07D239, C07D495, C07D333, C07D277, C07D335, C07D417, C07D403, C07D407, C07D413, C07D257, C07D209, C07D409, C07D417, C07D249, C07D295, C07D213, C07D305, C07D499, C07D307 | 1. Preservation of human body, animal and plant bodies or parts thereof; Biocides, e.g. as disinfectants, as pesticides or as herbicides; Plant growth regulator; 2. Steroid compounds; 3. [Heterocyclic compound](javascript:;). |
| 2 | C07H19, C07H15, C07H21, C08L31, C08L71, C12N15 | 1. Sugars and their derivatives, nucleosides, nucleotides, nucleic acids; 2. Compositions of high molecular compounds; 3. Mutation or genetic engineering; Genetic engineering involves DNA or RNA, vectors (such as plasmids) or their isolation, preparation, or purification. |
| 3 | A01H5, A01H6 | 1. New plants or methods of obtaining them; Plant regeneration through tissue culture techniques; |
| 4 | C12P7, C12P21, C12P19, C07C227, C07C303, C07C41, C07C67, C07K1, C08B37 | 1. Acyclic or carbonic compounds; 2. Fermentation or the use of enzymatic methods to synthesize target compounds or compositions or to separate optical isomers from racemic mixtures; 3. General preparation of peptides; |
| 5 | A01K67, A01N1, C12N5 | 1. Raising or breeding animals not included in other classes; New species of animals; 2. Preservation of human or animal bodies or parts thereof; 3. Undifferentiated human, animal, or plant cells, such as cell lines; Organization; Their cultivation or maintenance. |
| 6 | A61F13, A61F2, A61L15, A61G3, A61J3, A61M11, A61M15, A61M16, A61M35, A61M31, A61M1, A61M37, C03B7 | 1. An implantable blood vessel filter; Prosthesis; A device that provides openings for human tubular structures or prevents their collapse; A hot compress; Bandages, dressings or absorbent pads; 2. A device that injects a medium into or onto a human body; 3. Chemical aspects of bandages, dressings or absorbent pads; Or the material application of bandages, dressings or absorbent pads; 4. Ambulance aspects of vehicles; The vehicle has special equipment for the transport of the sick or disabled, or their special means of transport; 5. Fused glass dispenser; Tools for removing molten glass material; The manufacture of material drops; 6. A device or method for making a drug product into a particular physical or administrative form. |
| 7 | A61K31, A61K35, A61K39, A61K45, A61K48, A61K47, A61K38, A61K9, A61K51, A61K14, A61K33, A61K33, A61P1, A61P3, A61P9, A61P11, A61P13, A61P15, A61P17, A61P19, A61P21, A61P25, A61P27, A61P29, A61P33, A61P35, A61P37, A61P41, A61P43, A61Q17, A61Q19 | 1. A medical, dental, or grooming accessory; 2. The specific therapeutic activity of a compound or pharmaceutical preparation; 3. The specific use of cosmetics or similar toiletries. |
| 8 | C07F9, C07F7, B01D9, A01P1, C40B40, C30B7 | 1. An acyclic, carbon ring, or heterocyclic compound containing elements other than carbon, hydrogen, halogens, oxygen, nitrogen, sulfur, selenium, or tellurium; 2. Crystal; 3. Disinfectant; Antimicrobial compounds or their compositions; 4. The library itself, such as arrays, mixtures; 5. Solution of liquid solvent at room temperature. |
| 9 | G01N33, G01N1, G01N21, G01N27, G01N29, G01J3, A61L9 | 1. To test or analyze a material by measuring its chemical or physical properties; 2. Spectrometric method; Spectrophotometric method; A monochromator; Determination of color; 3. Disinfection, sterilization, or deodorization of air. |
| 10 | C07C69, C07C233, C07C235, C07C237, C07C257, C07C271, C07C215, C07C307, C07C309, C07C311, C07C317, C07C323, C07C333, C07C335, C07C35, C07C405, C07C43, C07C49, C07C62 | 1. Acyclic or carbonic compounds; |
| 11 | C07K2, C07K5, C07K7, C07K9, C07K14, C07K16, C07K19 | 1. Peptide; |
| 12 | C12N7, C12M1, C12N1, C12N13, C12N9, C12Q1, C12R1 | 1. A microorganism or enzyme; Propagation and preservation of microorganisms; genetic engineering; 2. Microorganism. |

**Table S3.** [Classification result](javascript:;).

| **[classification result](javascript:;)** | **Patent documents ( n= )** |
| --- | --- |
| treatment | 1156 |
| basic research | 234 |
| vaccine | 197 |
| others | 145 |
| testing | 59 |
| Total | 1791 |

**Table S4.** Country codes of patents by jurisdiction.

| **Abbreviation** | **Full name** |
| --- | --- |
| WO | World Intellectual Property Organization |
| US | United States of America |
| JP | Japan |
| CN | China |
| EP | European Patent Office |
| KR | Korea |
| AU | Australia |
| ES | Spain |
| BR | Brazil |
| CA | Canada |
| SG | Singapore |
| IL | Israel |
| TW | Chinese Taipei |
| MX | Mexico |
| DE | Germany |
| NZ | New Zealand |
| ZA | South Africa |
| NO | Norway |
| HK | Hong Kong (China) |
| IN | India |
| RU | Russian Federation |
| AR | Argentina |
| IE | Republic of Ireland |
| FI | Finland |
| DK | Denmark |
| SK | Slovakia |
| DD | Deutsche Demokratische |
| PT | Portugal |
| HU | Hungary |

1. **Supplementary figure**

**
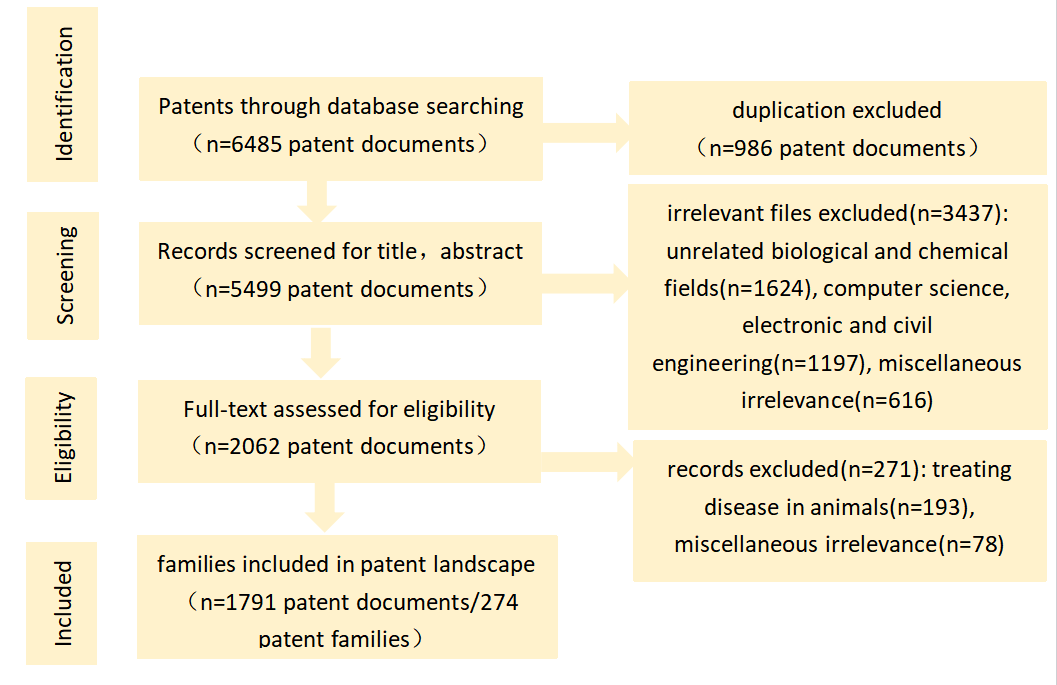
**

**Fig.S1** PRISMA flow diagram detailing the number of patent documents included at each stage and the reasons for removal.


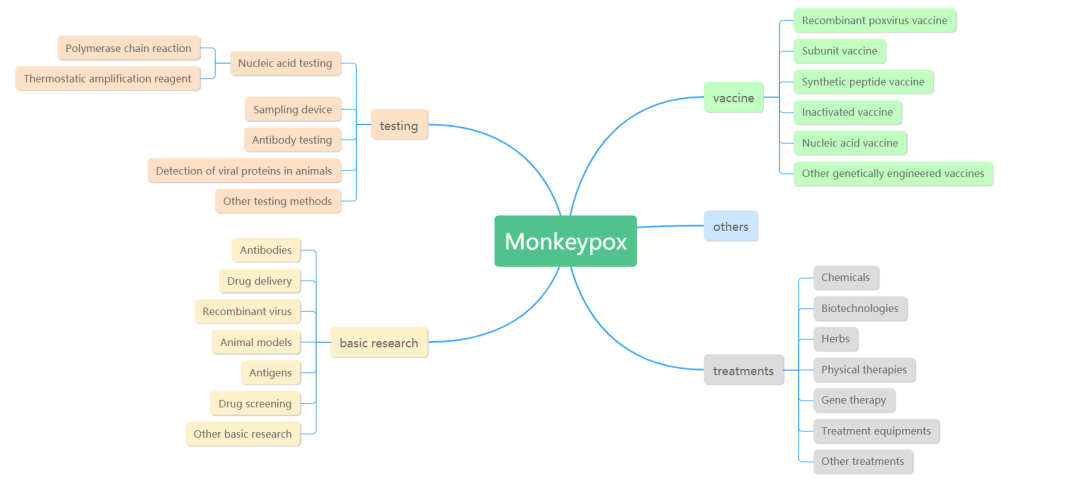


**Fig.S2** Classification process.


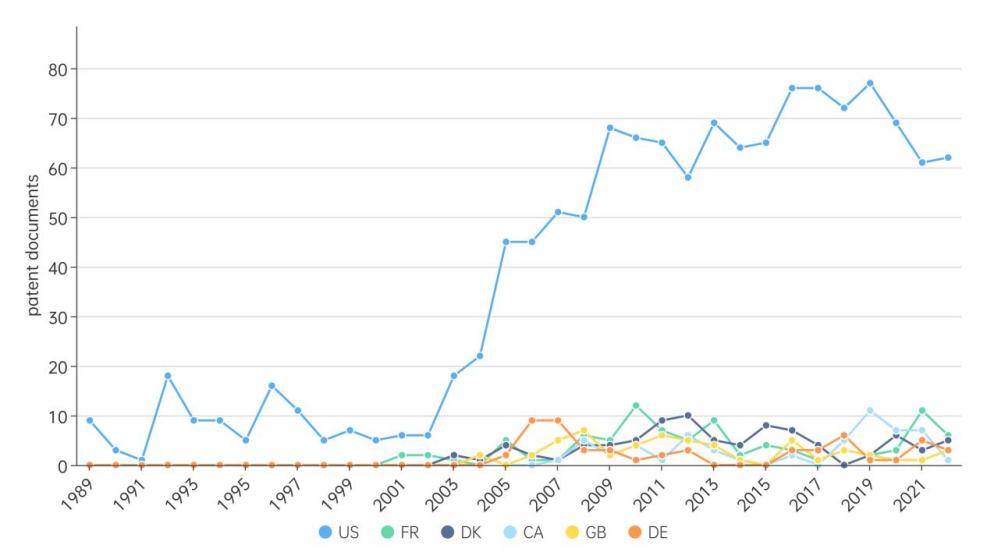


**Fig.S3** The temporal distribution of the patentee country and the “Two-Letter codes” by full country names are shown in Table S5.


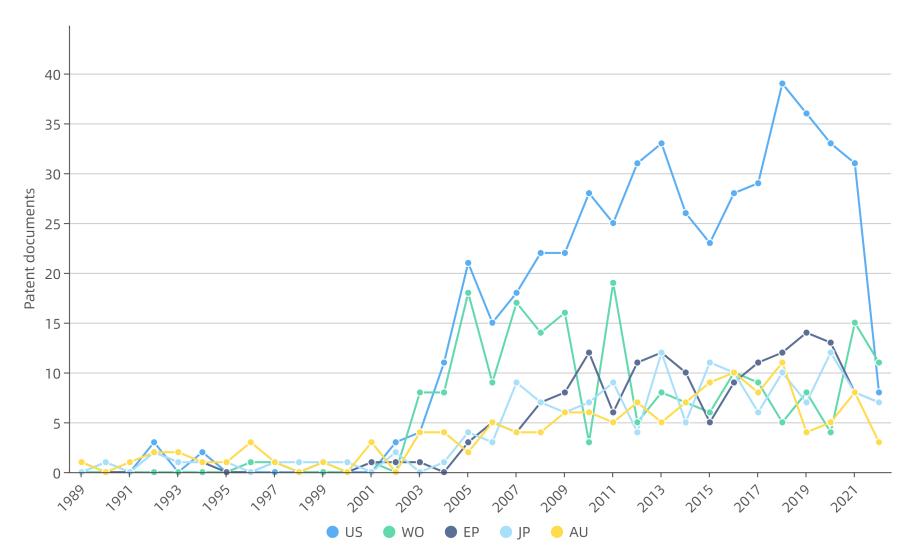


**Fig.S4** Time distribution of patent disclosure areas and the “Two-Letter codes” by full country names are shown in Table S5.
